# Meta-Cognitions in Tourette Syndrome, Tic Disorders, and Body-Focused Repetitive Disorder

**DOI:** 10.1177/070674371405900804

**Published:** 2014-08

**Authors:** Kieron O’Connor, Marie-Ève St-Pierre-Delorme, Julie Leclerc, Marc Lavoie, Mélodie T Blais

**Affiliations:** 1 Full Professor, Department of Psychiatry, University of Montreal, Montreal, Quebec; Director, OCD Spectrum Study Center, University Institute in Mental Health of Montreal, Montreal, Quebec.; 2 Doctoral Student, University of Quebec in Montreal, Montreal, Quebec.; 3 Professor, Department of Psychology, University of Quebec in Montreal, Montreal, Quebec; Associate Researcher, University Institute in Mental Health of Montreal, Montreal, Quebec.; 4 Associate Research Professor, University of Montreal, Montreal, Quebec; Director, Cognitive and Social Psychophysiology Laboratory, University Institute in Mental Health of Montreal, Montreal, Quebec.; 5 Doctoral Student, University of Montreal, Montreal, Quebec.

**Keywords:** cognitions, meta-cognitions, tic disorders, Tourette syndrome, body-focused repetitive behaviour

## Abstract

**Objective:**

To explore if self-reported presence of thinking about tics or body-focused repetitive behaviours (BFRBs; gests) are direct triggers of tic or gest onset in 3 groups: Tourette syndrome (TS; *n* =18), persistent chronic tic disorders (TDs; *n* = 42), and a comparison group with BFRB (*n* = 36).

**Method:**

The 3 groups completed a thinking about tics inventory, listing 22 items derived from clinician consensus that asked whether thoughts always, sometimes, or never exclusively triggered tic onset. Other questionnaires measured mood, perfectionism, impulsivity, premonitory urge, and self-rated tension. Sixty-three participants completed the inventory twice, and the inventory was completed pre- and post-behavioural intervention by a further 54.

**Results:**

The ranking of the thoughts reported as likely to trigger tics or gests was positively correlated across TD and BFRB groups. Exploratory principal components analysis of a reduced 12-item set (the thinking about tics inventory) in TS and TD groups revealed that such thoughts could be grouped into 3 separate subscales: thoughts about the interference of tics or gests, thoughts anticipating tics or gests, and thoughts about whether the person has permission to perform the tic or the gest. The 3 sets of subscales showed good and acceptable internal consistency and overall score showed good test–retest reliability, suggesting thoughts about tics or gests are robust and measurable. The subscales correlated with impulsivity, tic or behaviour severity, and ratings of frequency decreased post-behavioural treatment.

**Conclusions:**

Thinking about tics or gests is reported as triggering tics or gests in both TD and BFRB, and meta-cognition seems independent of premonitory sensations and relates to distinct clinical characteristics in each clinical group.

## Abstract (fr)

**Objectif:**

Explorer si la présence auto-déclarée de pensées sur les tics ou les comportements répétitifs centrés sur le corps (CRCC; gestes) est le déclencheur direct de l’apparition de tics ou de gestes dans 3 groupes : le syndrome de Tourette (ST; *n* = 18), les troubles tics chroniques persistants (TTs; *n* = 42), et un groupe témoin ayant des CRCC (*n* = 36).

**Méthode:**

Les 3 groupes ont rempli un inventaire de pensées sur les tics, composé de 22 items tirés d’un consensus de cliniciens qui demandaient si les pensées déclenchaient l’apparition des tics toujours, parfois, ou jamais exclusivement. D’autres questionnaires mesuraient l’humeur, le perfectionnisme, l’impulsivité, la pulsion prémonitoire, et la tension autoévaluée. Soixante-trois participants ont rempli l’inventaire deux fois, et 54 autres y ont répondu avant et après une intervention comportementale.

**Résultats:**

Le classement des pensées jugées susceptibles de déclarer des tics ou des gestes était positivement corrélé dans les groupes TT et CRCC. L’analyse exploratoire en composantes principales de l’inventaire réduit à 12 items (l’inventaire de pensées sur les tics) dans les groupes ST et TT a révélé que ces pensées pouvaient être groupées en 3 sous-échelles distinctes : les pensées sur l’interférence des tics ou gestes, les pensées anticipant les tics ou gestes, et les pensées à savoir si la personne a la permission d’exécuter le tic ou le geste. Les 3 ensembles de sous-échelles ont démontré une cohésion interne bonne et acceptable, et le score global indiquait une bonne fiabilité test-retest, ce qui suggère que les pensées sur les tics ou les gestes sont robustes et mesurables. Les sous-échelles étaient corrélées avec l’impulsivité, la gravité des tics ou des comportements, et les classements de la fréquence réduisaient le traitement post-comportemental.

**Conclusions:**

Selon ce qui est rapporté, penser aux tics ou aux gestes déclenche ceux-ci tant dans le TT que les CRCC. La métacognition semble indépendante des sensations prémonitoires et est liée aux caractéristiques cliniques distinctes de chaque groupe clinique.

Tics, as found in TS and chronic TDs, fluctuate in frequency, intensity, and location according to each person, and according to symptom evolution, in an individual way.^1^ Triggers for tic onset can include a range of state and situation factors including mood, stress, and feelings of incompleteness.^2^ Current thinking views tics as a neurodevelopmental disorder modifiable by contingency management. A behavioural therapy, CBIT remains an intervention of choice for tic management.^3^ However, tics also occur against a background of heightened sensory-motor activation and may be semi-voluntary responses to underlying psychophysiological tension or a sensory urge. Indeed, exposure and response prevention to the sensory urge has claimed equivalent efficacy to CBIT.^4^

There is growing evidence that cognitive processes, as well as chronic inner tension,^5^ are a background feature of tics and may be associated with certain TS-like characteristics.^6^ For example, perfectionist beliefs about action and organization are reported in TS and TDs.^7^,^8^ Anticipation of tics has also been reported as a trigger for tic onset.^9^ Talking or hearing talk about tics or viewing tics can facilitate tics,^10^,^11^ as these are behaviours that presumably shift attention to thinking about tics. Most people with TS report a premonitory sensation, a physical or mental urge, prior to tic onset.^12^ The complex nature of such premonitory signs preceding tic onset may include an interaction with cognitive attributions and meta-attentional focus.^9^ Do anticipatory vigilance or psychological awareness play a part in exacerbating or even creating premonitory urges? Although the influence of general tic-related beliefs on tic severity has been documented,^6^ so far there has been no systematic exploration of the role of meta-cognitive factors (thinking about tics) preceding tic onset.

BFRBs are a group of destructive and manual behaviours directed toward the body, including hair pulling (trichotillomania), skin picking, and nail biting, among others, and the person may repeat the complex manual action in an automatic or more focused manner.^13^ BFRBs are repetitive and voluntary,^13^ but clients with BFRBs report loss of control over the behaviour.^14^ BFRBs are heterogeneous and only recently recognized diagnostically.^1^

**Clinical Implications**

- People with both tic disorders and BFRBs report that thinking about the tic or gest produces onset.
- The thoughts about tics related separately to impulsivity and clinical severity and are modified following behaviour therapy.
- More attention should be paid to meta-cognitive, as well as behavioural and psychological, triggers to tics and BFRBs.

**Limitations**

- The findings were based on self-reported associations of thoughts at tic or gest onset.
- The PCA was exploratory and requires confirmation with a larger sample.

BFRBs form a suitable comparison group to TDs as the 2 disorders have similar psychological and emotional characteristics. For example, the urge to perform both BFRB and tics is often triggered by stimuli or situations. Like TS and TDs, there is a negative reinforcement cycle, and people with BFRB report short-term emotional relief post-repetitive behaviour (gest). But unlike most TDs, people with BFRBs do not generally report the premonitory sensory phenomena prior to the gest.^15^ Both groups respond to similar cognitive behavioural interventions.^16^

Our report aimed to present a preliminary clinical validation of an item-set measuring the perceived role of meta-cognition in tic onset. Meta-cognition was defined here as thoughts about performing the tic or the consequences or the appearance of the tic that the person perceived as triggering onset. The main objective was to first verify the existence of meta-cognitive thoughts in people with TS and TDs, and compare findings with the meta-cognition reported within a BFRB group prior to gest onset; second, to evaluate the extent to which meta-cognitions in these groups relate meaningfully to other clinical variables, such as tic severity, impulsivity, or self-reported tension; and third, to see whether such meta-cognitions are modifiable following CBT in all 3 groups.

## Method

### Instrument

The original item-set was composed of 22 items conceived by clinical consensus among clinicians at the OCD Study Centre in Montreal to measure thoughts, anticipation, and judgments likely to elicit a tic. The items were constructed from interviews with people with TDs identifying thoughts likely to produce tics. Psychological factors, such as awareness of the problem, reactions to feeling judged, or concerns about image, can trigger and inhibit tic or gests in BFRB. The attempt to suppress a tic can generate the urge as can suggestion, where thinking about tic onset, or observing another person’s tics seem to elicit the tic. Such suggestibility can be noticeable in a group setting where one person’s tic may facilitate the appearance of other people’s tics. Items included simple anticipation of a tic, thoughts about the interference of a tic, the social consequences of a tic, about the reaction of others to tics, the impact of thoughts on self-image, and of feelings that it is permissible or not to tic. All items were phrased as positive questions as the concept being measured was thoughts perceived as eliciting tics or habits, not a dimension of noneliciting, compared with eliciting thoughts. The instructions asked the participant to indicate whether, in their experience, the following thoughts had triggered tic or gest onset prior to any sign. Responses were coded 1 to 3, denoting always (1), sometimes (2), or never (3). The study was approved by the local institutional ethics board and all participants gave informed consent.

### Participants

The recruitment was through the OCD Spectrum Study Centre in Montreal, and participants were 96 consecutive Francophone referrals diagnosed with TS (18), chronic TD (42), and tic group or BFRB (36) as a primary diagnosis. The mean age was 38.47 years (SD 12.49), with 58% (49) being female. The TS-TD sample consisted of 26 simple tics and 34 complex tics. Mean age of onset was 11.2 years (SD 1.3). In the BFRB sample, the comparison group consisted of hair pulling (*n* = 19), nail biting (*n* = 8), skin picking (*n* = 7), and bruxism (*n* = 2). Mean age of onset was 14.3 years (SD 2.0). Among the TS-TD group, 31 were single, 24 were university educated, and the rest received secondary education or below; and of the BFRB group, 13 were single and 18 had received a university education, and the rest had received secondary education or below. There were no differences between the TS-TD, and BFRB groups on any demographic variables (*P* > 0.30), but the TS-TD group contained more females (33, compared with 7 males; *P* < 0.01). Twenty-one per cent of the sample were taking antidepressants, 6% were taking anxiolytics, 2% were taking antipsychotics, and 4% were taking neuroleptics. Some participants were taking part in an open treatment trial; 56% (*n* = 54) were also assessed posttreatment. Also, 63 (11 TS, 31 TD, and 21 BFRB) participants completed the item inventory twice, one month apart, at pretreatment to check coefficients of test–retest reliability (Table 1).

The BAI^17^ was administered and consists of a 21-item anxiety symptom checklist, rating symptom intensity for the last week on a 0-to-3 scale. Its total score ranges from 0 to 63. The French version of the BAI shows good psychometric properties (internal consistency, test–retest stability, and convergent and divergent validity).^18^

The BDI-II^19^ consists of a 21-item relative to depression. The BDI assesses cognitive, emotional, and somatic depressive symptoms on a 0-to-3 scale. Its total score ranges from 0 to 63. The French version of the BDI shows good psychometric properties (internal consistency and test–retest reliability).^20^

The Frost MPS^21^ is a self-administered questionnaire of 35 items (1 to 6) covering 6 dimensions: concern over mistakes; personal standards; parental expectations; parental criticism; doubts about actions; and importance of organization and order. The coefficient of internal consistency is satisfactory, with good test–retest reliability. Total score ranges from 35 to 175 (French validation^22^).

The BIS-10^23^ is one of the oldest and most widely used measures of impulsive personality traits on a 4-point scale (1 to 4). It includes 34 items measuring attention, motor, impulsiveness, and nonplanned impulsiveness. A total score ranges from 34 to 136. The ICC for the total score is good (French validation^24^).

The Self-Rated Tension Scale is a 12-item, clinical scale asking numerous questions about participants’ degree of tension, with a response of true or false to each item (score range 0 to 12). Items include: “I am conscious of being more tense than necessary”; “Others have remarked on my being tense”; “I don’t seem to be able to function without tension”; and “Tension restricts my activities.” The ICC French version is good (α = 0.72).

Participants were screened by telephone and assessed face-to-face by the Structured Clinical Interview for the Diagnostic and Statistical Manual of Mental Disorders^25^ by trained evaluators. Criteria for inclusion were as follows: presenting a simple or complex tic or gest for at least 1 year occurring daily. Participants included in the TS group were age 18 to 65 years and had a diagnosis of TS as the principal presenting problem accompanied by vocal tics. People included in the TD group presented a simple or complex tic (vocal or motor) for at least 1 year occurring daily. Criteria for exclusion were as follows: any major medical history, head injury including sensory-motor impairment, history of autism, and an IQ of less than 75; other psychiatric problems on Axis I or II (including comorbidity of OCDs), any neurological problems (for example, Parkinson disease, hemifacial spasms, Meige syndrome, sclerosis, Huntingdon disease, and Wilson disease); currently receiving treatment from a psychologist, acupuncturist, hypnotherapist, or a massotherapist; and currently receiving psychotropics nonrelevant to TS or attention-deficit hyperactivity disorder or abuse of alcohol or drugs. TS symptom severity was assessed with the TSGS^26^ and the YGTSS.^27^ Both questionnaires were administered by the clinician through a structured interview. The TSGS is the older tic symptom rating scale and has increased sensitivity to multidimensional change. Participants were also assessed on the OC-TS semi-structured interview, which poses questions on history, onset, form, location, and nature of the tic or habit gest, and the presence, nature, and duration of any premonitory sensory urge.^28^ BFRBs were assessed, respectively, with the Massachusetts Hair Pulling Scale,^29^ Massachusetts Skin Picking Scale,^30^ and similar scales adapted for the specific BFRB, as well as on the TSGS. It is also feasible to assess BFRB using the TSGS (see Assessment of BFRBs Using TSGS below), and BFRB was assessed identically to tics in terms of frequency, severity, and form of occurrence on the TSGS and OC-TS. Inclusion criteria for the BFRB group were as follows: age 18 to 65 years; current BFRB with a subjective severity rating of at least 3/10, and significant distress or impairment from BFRB; BFRB as the primary presenting problem, even if another psychological problem or disorder was present; and if on psychotropics, medication had to be stabilized for 3 months. The first TSGS subscale rated the nature of the tic (that is, motor or phonic), while the second scale rated the tic complexity. A third scale assessed functional impairment, including behavioural, learning, motor restlessness, and occupational problems. According to past research,^27^ the inter–rater reliability of the TSGS global score was found to be very good (κ = 0.77, *P* < 0.001). Convergent validity of the motor and phonic tic factors is shown by strong correlations between the TSGS and the YGTSS (*r* = 0.86 to 0.91).

Diagnosis was verified in 25% of cases selected at random by an independent rater. In all cases, there was 100% interrater agreement between initial and subsequent diagnosis.

## Results

### Response Variability

The scores for the 22 items for both the TS-TD and BFRB groups are given in Table 2. It can be seen that the items rated most likely to trigger tics or gests are similar in both the TS-TD and BFRB groups. A Spearman rho correlation coefficient of rankings between the TS-TD and BFRB groups was highly significant (*r*s = 0.68, *df* = 22, *P* < 0.001). Response distribution to individual meta-cognitive items was examined through a histogram, which showed that 19 items had a normal symmetric distribution of scores across the range of response options (1 to 3) in TS-TD and BRFB groups. There was no item where all participants responded never or all participants responded always, indicating a parametric range of individual differences, with no skewness or kurtosis.

As responses were similar in both groups, and to give some structure to the remaining items, responses were subjected to PCA, with oblimin rotation, in a pooled sample (*n* = 96) of TS-TD and BRFB groups. Two items with low communality coefficients were eliminated. A further 7 items, with low loadings (less than 0.40), were eliminated.

A PCA on the reduced 12-item scale, with oblimin rotation and Kaiser normalization, was then computed in the tic group only (*n* = 60). The ratio of variables to factors was about 5:1, which, depending on authorities, is an acceptable ratio, especially with high communalities.^31^ Three factors emerged with Eigen values of less than 1.0 and accounting for 63.4% of the variance, with the 3 factors, respectively, accounting for 38.6%, 13.7%, and 11.1% of the variance. The 3-factor extraction was confirmed by parallel analyses.^32^ Correlation among the factors was significant but low.

The first factor was labelled “interference” (the dominant theme of this factor was thinking that a tic would negatively impact an activity or self-image). The second factor was labelled “anticipation,” and was characterized by thinking about tics or anticipating a tic. Interestingly, the item “noticing that you have not ticced” loaded high on this second factor. The third factor was labelled “permission,” and reflected thoughts that the person had permission to tic. The final 3-factor, 12-item scale is shown in Table 3. The total item score should be interpreted as an overall index of the perceived relevance of thoughts to tic onset.

### Internal Consistency

Cronbach alpha measures of ICC were, respectively, as follows: scale 1 (interference): 0.88; scale 2 (anticipation): 0.79, both of which were good; and scale 3 (permission): 0.65, which is acceptable. The Cronbach alpha for all 12 items was 0.86.

### Reliability

Test–retest reliabilities were calculated on a subsample of 63 participants who completed the questionnaire twice, one month apart, and were for Factor 1 (interference) = 0.75; Factor 2 (anticipation) = 0.78; and Factor 3 (permission) = 0.56, for an overall reliability of 0.70. All test–retest coefficients for the 12 items were significant beyond the *P* < 0.02 level.

### Assessment of BFRBs Using TSGS

BFRBs were assessed with appropriate instruments (see Clinical Assessment participants) but to permit direct comparison of clinical severity between TD and BFRB groups, the BFRB were additionally assessed using the TSGS by changing the word tic for the specific BFRB gest. The feasibility of this method of scoring BFRB was supported by a correlation of the TSGS tic rating with the appropriate BFRB scale frequency rati ng (nail biting and skin picking [*r* = 0.52, *df* = 15, *P* < 0.05]; or hair pulling [*r* = 0.49, *df* = 18, *P* < 0.04]).

### Group Differences

Analysis of variance tested for group differences in factor scores. There was a significant difference between TD and the BFRB group on the second anticipation subscale pretreatment, where the TS and TD groups rated the thoughts more likely on this subscale, whereas the BFRB group rated the thoughts less likely (*F* = 8.60, *df* = 1/94, *P* < 0.004). The BFRB group also scored thoughts related to permission significantly less likely to trigger gests on the third permission subscale (*F* = 6.88, *df* = 1/94, *P* < 0.01). There were no group differences on the first interference subscale.

### Convergent or Divergent Validity

Pearson product–moment correlations were calculated pretreatment between subscale scores and tic symptomatology, mood, and other psychosocial instruments. After Bonferroni correction, the significance level was set at *P* < 0.01, but trends (*P* < 0.05) are reported. For both groups, subscales 2 (anticipation) and 3 (permission) correlated significantly with TSGS tic severity (*r* = −0.23, *df* = 94, *P* < 0.02; *r* = −0.25, *df* = 94, *P* < 0.01) and with the BIS-10 (*r* = −0.30, *df* = 94, *P* < 0.003; *r* = −0.29, *df* = 94, *P* < 0.004). The correlations remained significant (*P* < 0.01) after partialing out the BIS-10. The BIS-10 correlated with the Self-Rated Tension Total Scale (*r* = 0.26, *df* = 94, *P* < 0.01). The higher tic severity, the more frequent thoughts about tics were experienced and the higher the BIS-10 and Self-Rated Tension Total Scale. In the tic (TD-TS) group, the total 12-item score correlated with the total score of the BIS-10 (*r* = −0.44, *df* = 58, *P* < 0.001). Subscale 2 (anticipation) and 3 (permission) also correlated with the total BIS-10 score (anticipation: *r* = −0.37, *df* = 58, *P* < 0.004; permission: *r* = −0.45, *df* = 58, *P* < 0.001). There was also a trend to correlation between the permission subscale and Self-Rated Tension Total Scale score (*r* = −0.25, *df* = 58, *P* < 0.05). There were no significant correlations of the subscales or total scores with the YGTSS.

In the BFRB group, there was no correlation of the total or subscale scores with impulsivity. The interference subscale also correlated strongly with the total TSGS score (*r* = −0.51, *df* = 34, *P* < 0.001). In the BFRB group, the anticipation subscale correlated significantly with the Self-Reported Tension Scale (*r* = −0.46, *df* = 34, *P* < 0.004).

### Clinical Validity

Pre–post change in subscale scores was calculated for 54 participants after they had received 14 weeks of CBT. The CBT was a 10-step adapted version of habit reversal (the cognitive psychophysiological model),^9^ where TS-specific processes involving motor planning and style of action, and beliefs about tics are addressed, and competing responses are integrated into cognitive-behavioural restructuring.^33^ Both tics and BFRBs were scored using the TSGS. For the entire sample (*n* = 54), tic symptoms, as measured by total TSGS score, reduced significantly from TSGS 15.37 to 7.26. The total change pre–post for all 12 items of the THAT was significant (*F* = 11.77, *df* = 1/53, *P* < 0.001). The mean scores on scale 1 (interference) decreased pre–post significantly (*F* = 18.97, *df* = 1/53, *P* < 0.001). The separate TS-TD (*n* = 30) and BFRB (*n* = 24) groups change posttreatment are given in Table 4. The BFRB group showed less decrease posttreatment than the TD group. Here the TS-TD group showed a significant decrease in the interference subscale (*F* = 6.20, *df* = 1/29, *P* < 0.02) and the anticipation subscale (*F* = 5.90, *df* = 1/29, *P* < 0.02), and the BFRB group showed a significant decrease in the interference subscale (*F* = 4.36, *df* = 1/23, *P* < 0.05).

The OC-TS structured interview asks questions about the presence and nature of premonitory sensations. Fourteen per cent of the TS group reported no premonitory sensation, and all but one of the other 86% of the sample reported the sensation as physical (rather than mental). Sixteen per cent of the TD group reported no sensation, and again the other 84% except one person reported the sensation as physical. Thirty-three per cent of the BFRB group reported experiencing no premonitory sensation, and of the 67% who did experience some urge, 63% reported the premonitory urge was mental rather than physical. The findings seem to substantiate differences in the experience of urges between the TS-TD and BFRB groups.

## Discussion

Our study aimed to see whether tic and BFRB participants reported the presence of meta-cognitions prior to tic or gest onset and whether the frequency varied across the TS-TD and BFRB samples and whether self-report meta-cognitions related to clinical features and were modifiable post-CBT. The results from THAT item-set and the exploratory validation of the item-set testify to the perceived role of thoughts about tics in eliciting tics, a notion previously noted anecdotally in clinical practice. Both the TS-TD and BFRB groups showed significantly related rankings of the thoughts likely to trigger tic occurrence. It seems several types of thoughts are perceived as likely to provoke tics. The subscales of the THAT item-set included the following thoughts: that tics may occur, that they may negatively impact on activities or self and appearance, and whether or not the person felt they were allowed or not to tic. Respondents reported that all 3 types of thinking showed the potential to influence or inhibit tics. Two of the 3 subscales of the THAT had good test–retest reliability and ICC, thus demonstrating that such thoughts are quantifiable in tic and BFRB populations. In other words, thinking about tics is perceived to relate directly to tic onset. Also, thinking about tics did not correlate with mood variables, such as anxiety or depression or to perfectionism, but did relate to impulsivity in TS-TD and more definitively to tic severity in BFRB.

Thoughts about tics leading to tic onset in TS-TD may form part of poor impulse control in people with TDs and hence correlate with impulsivity, although it did not seem mediated by impulsivity. An urge to perform the BFRB gest seems more cognitive than physically present in the BFRB group, and here the thoughts about the gest related to clinical severity but not impulsivity. The anticipation subscale was also significantly associated with self-reported muscle tension. However, causality is unclear: does more anticipation bring about more tension or vice versa? The correlation of the Self-Rated Tension Total Scale with higher frequency of thoughts may suggest that increased muscle contraction is associated with thoughts, and there may be a tri-directional classic triangle linking thoughts, chronic tension, and tic onset.

Two subscales (interference and anticipation) of the THAT showed reduced scores post-CBT in the TS-TD group, and the interference scale significantly reduced post-CBT treatment in the BFRB group, suggesting the thoughts are modifiable by behavioural intervention or that the thoughts change as a consequence of reduction in tics or behaviour following CBT.

There are limitations to our study. The findings were based on self-reported associations of thoughts with tic onset. The PCA aim was exploratory and was intended rather to structure the item-set for the purposes of comparing groups and examining clinical convergence. However, our results are promising: minimal criteria for PCA were met and the present psychometric properties and findings on the item-set were robust. Further, the presence of high communalities, a small number of factors, no cross loadings, and several variables loading on the factors, strengthens PCA with a small sample size.^31^ A confirmatory factor analysis of the THAT would complement the exploratory PCA. Future research may investigate the role of thoughts in children and other age groups, and concretely explore the situations in which each of the different types of thoughts are likely to occur, using experimental manipulation of thoughts while monitoring tic onset.

## Figures and Tables

**Table 1:** TSGS symptom severity, questionnaire scores, and THAT (subscale and total) scores from combined TS-TD and BFRB groups at baseline

Measures	TD-TS, *n* = 60	BFRB, *n* = 36
	Pre (SD)	Pre (SD)
Symptom severity		
Total	18.38 (11.78)	12.81 (9.15)
Tic	10.27 (7.14)	4.98 (3.38)
Behaviour	8.11 (7.31)	7.82 (7.42)
Beck Anxiety Inventory	9.66 (9.32)	9.11 (7.14)
Beck Depression Inventory	8.02 (6.73)	7.01 (7.09)
Barratt Impulsiveness Scale-10	75.30 (6.14)	74.56 (5.95)
Multidimensional Perfectionism Scale	97.13 (24.19)	99.08 (21.64)
THAT^a^		
Total	2.08 (0.44)	2.26 (0.49)
Interference	2.10 (0.63)	2.11 (0.55)
Anticipation	1.98 (0.51)	2.28 (0.40)
Permission	2.15 (0.50)	2.40 (0.36)

a 1 = always; 2 = sometimes; 3 = never

BFRB = body-focused repetitive behaviour; TD = tic disorder; THAT = Thinking About Tics Inventory; TS = Tourette syndrome; TSGS = Tourette Syndrome Global Scale

**Table 2:** Thinking About Tics Inventory

Please indicate whether any of the following thoughts have triggered your tics:	TS-TD (*n* = 60) Mean (SD)	BFRB (*n* = 36) Mean (SD)
Anticipating that you might tic	1.85 (0.68)	2.08 (0.69)
Thinking in general about your tics	1.85 (0.63)	2.06 (0.62)
Thinking others will observe you ticcing	1.95 (0.74)	2.39 (0.68)
The idea that you must tic to feel relief	1.82 (0.65)	1.92 (0.73)
Talking about your tics	2.03 (0.71)	2.42 (0.50)
Knowing you will be with people who expect you to tic	2.30 (0.67)	2.58 (0.60)
Wondering if your tics will interfere with your activities	2.15 (0.70)	2.33 (0.67)
Asking yourself if you will always have tics	2.15 (0.70)	1.94 (0.67)
Asking yourself whether your tics will get worse	2.25 (0.65)	2.08 (0.69)
Knowing you have permission to tic	2.17 (0.76)	2.39 (0.68)
Knowing that other people do not have tics like you	2.43 (0.72)	2.58 (0.64)
Thinking you will need to suppress a tic	1.80 (0.73)	1.97 (0.65)
Knowing you should not be ticcing	1.85 (0.73)	1.97 (0.65)
Wishing you did not have tics	2.05 (0.74)	2.00 (0.71)
Thinking tics spoil your image	2.07 (0.75)	2.42 (0.50)
Thinking you appear odd and different due to your tics	2.07 (0.75)	2.11 (0.74)
Dwelling on how your tics tire you out	2.10 (0.70)	2.31 (0.62)
Finding your tics annoying or distressing	1.83 (0.69)	1.81 (0.62)
Seeing yourself tic	1.82 (0.70)	1.86 (0.68)
Observing someone else tic	2.30 (0.78)	2.69 (0.46)
Thinking your tics make you look bad	2.13 (0.74)	2.03 (0.69)
Noticing you have not ticced for some time	1.95 (0.64)	2.31 (0.62)

**Table 3:** TSGS symptom severity and THAT total score and subscale means pre- and posttreatment for 30 TS-TD and 24 BFRB participants who received behavioural treatment showing significant changes

Measures	TD-TS		BFRB	
	Pre (SD)	Post (SD)	Pre (SD)	Post (SD)
Symptom severity				
Total scale	18.33 (11.48)	8.15 (7.79)^a^	13.59 (7.89)	6.08 (7.00)^a^
Tic scale	10.08 (6.51)	4.65 (5.09)^a^	5.19 (2.73)	2.40 (1.66)^a^
Behaviour scale	8.24 (7.66)	3.57 (3.97)^b^	8.40 (6.63)	4.58 (6.04)^a^
THAT^c^				
Total scale	2.07 (0.39)	2.46 (0.33)^a^	2.28 (0.32)	2.46 (0.35)
Interference subscale	2.06 (0.53)	2.51 (0.40)^b^	2.10 (0.57)	2.46 (0.51)^b^
Anticipation subscale	1.99 (0.47)	2.41 (0.36)^b^	2.30 (0.44)	2.30 (0.38)
Permission subscale	2.18 (0.50)	2.47 (0.33)	2.45 (0.38)	2.44 (0.38)

a *P* < 0.001;

b *P* < 0.05;

c Scores: 1 = always; 2 = sometimes; 3 = never

BFRB = body-focused repetitive behaviour; TD = tic disorder; THAT = Thinking About Tics Inventory; TS = Tourette syndrome; TSGS = Tourette Syndrome Global Scale

**Table 4:** 12-item loadings on the 3 scales of Thinking About Tics Inventory

Item number	Item	Interference 1	Anticipation 2	Permission 3
1	Anticipating that you might tic		0.76	
2	Thinking in general about your tics		0.86	
4	The idea that you must tic to feel relief			0.56
5	Talking about your tics		0.65	
6	Knowing that you will be with people who expect you to have tics			0.57
7	Wondering if your tics will interfere with your activities	0.76		
10	Knowing that you have permission to tic			0.84
15	Thinking that the tics spoil your image	0.86		
16	Thinking that you appear odd and different due to your tics	0.82		
20	Observing someone else tic			0.60
21	Thinking that your tics make you look bad	0.87		
22	Noticing that you have not ticced in a while		0.75	

## Abbreviations

BAI: Beck Anxiety Inventory
BDI: Beck Depression Inventory
BFRB: body-focused repetitive behaviour
BIS: Barratt Impulsiveness Scale
CBIT: comprehensive behavioural intervention for tics
CBT: cognitive-behavioural therapy
ICC: internal consistency coefficient
MPS: Multidimentional Perfectionism Scale
OCD: obsessive–compulsive disorder
OC-TS: OCD-Tourette Syndrome Scale
PCA: principal component analysis
TD: tic disorder
THAT: Thinking About Tics Inventory
TS: Tourette syndrome
TSGS: Tourette Syndrome Global Scale
YGTSS: Yale Global Tourette Syndrome Scale
